# Symptoms and quality of life in gynecological cancer patients after surgery: Application of latent profile and network analysis

**DOI:** 10.1097/MD.0000000000049482

**Published:** 2026-06-26

**Authors:** Wenting Liu, Juan He, Han Chen, Haiyan An, Lu Jiang

**Affiliations:** a Second Department of Gynecology, The First Affiliated Hospital of Xinjiang Medical University, Urumqi, Xinjiang, China; c First Department of Gynecology, The First Affiliated Hospital of Xinjiang Medical University, Urumqi, Xinjiang, China; b Second Department of Oncology, The First Affiliated Hospital of Xinjiang Medical University, Urumqi, Xinjiang, China

**Keywords:** core symptoms, gynecological malignancies, latent profile analysis, quality of life, symptom network

## Abstract

This study aimed to identify latent classes of postoperative symptoms in patients with gynecological malignancies and analyze the complex relationship between different symptom categories and quality of life (QoL). A convenience sample of 385 postoperative patients with gynecological malignancies was surveyed using a general information questionnaire, the Chinese version of the MD Anderson Symptom Inventory for Gynecological Cancer, and a QoL scale. Symptoms with an incidence >20% were subjected to latent profile analysis to identify symptom subgroups. Network analysis was used to construct symptom– QoL networks for each subgroup. Patients were categorized into a low symptom burden group (67.5%) and a moderate-to-high symptom burden group (32.5%). Age, preoperative chemotherapy, and cancer type were factors significantly associated with symptom burden. In the low burden group, physical symptoms were core features, with emotional status serving as bridge symptom. The moderate-to-high burden group exhibited a clearer “psychological-somatic” comorbidity pattern, with physical status acting as a bridge symptom. Significant heterogeneity exists in early postoperative symptoms (assessed on postoperative day 3) among gynecological malignancy patients. Interventions for the low symptom burden group should focus on physical symptoms, while emotional regulation is central to symptom management in the moderate-to-high burden group. Tailored symptom management strategies based on subgroup characteristics may help improve patients’ QoL during the acute recovery period.

## 1. Introduction

The global incidence of common gynecological malignant tumors (such as cervical cancer, endometrial cancer, and ovarian cancer) has been continuously increasing, with approximately 1424,000 new cases reported in 2022.^[1]^ Surgery is one of the main treatment modalities, and patients experience a series of complex postoperative symptoms,^[2]^ including fatigue, pain, distress, abdominal distension, and others. These symptoms often occur concurrently, forming symptom clusters, which severely impair patients’ physical function, psychological status, and quality of life (QoL).^[3]^ Currently, symptom management research is shifting from a population-level perspective to focusing on individual differences. Miaskowski et al^[4]^ emphasized that identifying patient subgroups with distinct symptom experiences is critical for achieving precise symptom management. Existing domestic studies have mostly been limited to population-level analyses of symptoms,^[5]^ with few exploring potential subgroups within the population. Such neglect of heterogeneity may reduce the effectiveness of symptom management. Latent Profile Analysis (LPA) is an individual-centered approach that can identify latent subgroups with similar presentation patterns based on symptom characteristics.^[6]^ Network analysis can further reveal the relationships among symptoms and between symptoms and QoL, as well as identify core and bridging symptoms.^[7]^ Therefore, this study integrates LPA and network analysis to identify symptom subgroups among postoperative patients with gynecological malignant tumors and the network characteristics of symptoms and QoL within different subgroups, thereby providing a basis for the formulation of precise symptom management strategies.

## 2. Methods

### 2.1. Participants

A convenience sampling method was used in this study. This study employed a cross-sectional design. Patients were enrolled if they underwent surgical treatment for gynecological malignant tumors at a tertiary Grade A hospital in Xinjiang between September 2022 and September 2024.

Inclusion criteria:

1. Patients pathologically diagnosed with cervical cancer, ovarian cancer, or endometrial cancer;
2. Aged ≥18 years;
3. Aware of their own disease condition and diagnosis;
4. Possessing verbal communication and comprehension abilities, having signed informed consent, and voluntarily participating in the study.

Exclusion criteria:

1. Presence of mental or consciousness disorders;
2. Complicated with other malignant lesions or life-threatening diseases, poor general condition, and unable to cooperate with the investigation.

The study protocol was approved by the Ethics Committee of The First Affiliated Hospital of Xinjiang Medical University (Approval No.: K202208-45).

### 2.2. General information questionnaire

A general information questionnaire was independently designed by the investigators based on a literature review, covering sociodemographic characteristics including age, occupation, education level, marital status, living arrangement, place of residence, source of medical expenses, and whether they had children; disease-related data including surgical approach, type of disease, and receipt of preoperative chemotherapy.^[8]^

### 2.3. Chinese version of the MD Anderson symptom inventory–perioperative gynecologic cancer (MDASI-PeriOp-GYN)

This scale was developed by the MD Anderson Cancer Center in 2018 and was subsequently translated, adapted, and revised into Chinese by Zheng Yingying et al.^[9]^ It consists of a gynecologic cancer-specific module (9 symptoms) and a general cancer module (13 symptoms). Symptom severity was rated on an 11-point scale from 0 to 10, ranging from “no symptom” to “worst imaginable severity,” with a total score ranging from 0 to 220. Higher scores indicate a greater perioperative symptom burden. The Cronbach’s α coefficient of the Chinese version was 0.798 (22 items in total), and the item-level content validity index of each item was higher than 0.71, indicating good reliability and validity for clinical empirical research.

### 2.4. Chinese version of the functional assessment of cancer therapy-general

The scale was developed by the Center on Outcomes, Research, and Education at Northwestern University.^[10]^ It contains 27 items divided into 4 domains: physical well-being, social/family well-being, emotional well-being, and functional well-being. All items were scored on a 5-point Likert scale. Items 1 to 7 of physical well-being and items 1, 3 to 6 of emotional well-being were reverse-scored, while the others were forward-scored. Higher Functional Assessment of Cancer Therapy-General scores (0–108) represent a better QoL. The scale has been applied in more than 50 countries worldwide. Its Chinese version was validated by Yu et al^[11]^ with good reliability and validity, and is suitable for QoL assessment in cancer patients.

### 2.5. Data collection and quality control

To ensure consistency of the questionnaire, all questionnaires were distributed by 3 uniformly trained investigators to standardize the procedure. According to reference^[12]^ and results of a preliminary survey among clinical patients, patients exhibited prominent physical and psychological symptoms on postoperative day 3 and maintained adequate capacity and willingness to complete questionnaires. This time point was selected because symptom severity often peaks within the first 72 hours after surgery,^[13,14]^ offering a clinically informative window for evaluating acute symptom burden and initiating early interventions. Of note, symptom profiles during this period also reflect acute physiological conditions, including systemic inflammatory responses and opioid-related effects.

For patients meeting the inclusion criteria, researchers fully explained the purpose, content, and significance of the study. Patients were enrolled after providing written informed consent. Questionnaires were collected and checked on-site. A total of 400 questionnaires were distributed, and 385 valid questionnaires were recovered, yielding a valid response rate of 96.25%.

### 2.6. Statistical analysis

Symptoms with an incidence rate higher than 20% were included in the LPA. This threshold was established a priori for 3 reasons. First, simulation studies demonstrate that indicators with low endorsement rates yield severely skewed, zero-inflated distributions that can increase boundary estimates, reduce class separation, and compromise model convergence.^[15,16]^ Excluding such symptoms enhances the stability and reliability of the latent class solution. Second, expert consensus on symptom cluster research advises that variable selection should balance clinical significance with statistical feasibility.^[4]^ Third, prevalence-based inclusion criteria have been adopted in several LPA studies of cancer‑related symptoms^[17]^; the 20% cutoff is consistent with this body of work and facilitates cross‑study comparability. No data imputation was performed, and a complete‑case analysis was used. Data were entered using SPSS 27.0. LPA was performed using Mplus 8.3, and symptom–QoL networks were constructed using R 4.5.1. Model fit was evaluated based on the following criteria^[18]^: Information criteria, including the Akaike information criterion, Bayesian information criterion, and sample size-adjusted BIC; lower values indicate better model fit. Bootstrapped likelihood ratio test and Lo-Mendell-Rubin test; a significant *P*-value indicates that the model with K classes is superior to the model with K-1 classes. Entropy: values closer to 1 indicate clearer class separation and higher classification accuracy. LPA was conducted using robust maximum likelihood estimation, which adjusts standard errors and test statistics for non-normality. The symptom severity distributions were skewed and zero-inflated. Observations were independent, with each patient assessed once.

For network analysis, the bootnet package was used to construct symptom–QoL networks, and the qgraph package was used for network visualization. For each subgroup, the network was estimated using the EBICglasso algorithm, which applies L1 regularization (graphical lasso) and selects the model by minimizing the Extended Bayesian Information Criterion with the tuning parameter *γ* = 0.5. Networks were plotted using the Fruchterman–Reingold force-directed algorithm, placing nodes with the strongest correlations at the center of the network.^[19]^ Among centrality indices, node strength was used to identify core symptoms,^[20]^ and bridge strength was used to identify bridge symptoms.^[21]^ Network stability was assessed using the correlation stability coefficient (CS-coefficient) for strength centrality. CS-coefficient values above 0.25 indicate acceptable stability, and values above 0.50 indicate good stability. Bootstrapped difference tests for edge weights, strength, and bridge strength were performed to evaluate the precision of the estimated network connections and the reliability of centrality differences.

Categorical variables were coded as factors with specified reference categories: age (≥60 years as reference), preoperative chemotherapy (no as reference), and disease type (cervical cancer as reference). Potential confounders were selected based on clinical relevance and univariate significance (*P* < .05); age, preoperative chemotherapy, and disease type were entered into the multivariable model. Multicollinearity among the independent variables was assessed using the generalized variance inflation factor (GVIF). GVIF^ (1/ (2 × df)) values close to 1 indicate no concerning multicollinearity; the conventional threshold for detecting multicollinearity is VIF >5.^[22]^ Model fit was evaluated using the Hosmer–Lemeshow goodness-of-fit test and the area under the receiver operating characteristic curve (AUC). Firth penalized logistic regression was performed as a sensitivity analysis to evaluate model stability and confirm that estimates were robust against separation bias^[23]^; detailed results are presented in Supplementary Table S2, Supplemental Digital Content 1.

## 3. Results

### 3.1. Study population characteristics

Of the 400 questionnaires distributed, 15 were excluded due to incomplete responses, yielding a final analytic sample of 385. No variables in the 385 valid questionnaires had missing values. This study enrolled 385 patients, predominantly (85.46%) aged 40 years or older, and the majority (92.21%) were married. The distribution of cancer types included cervical cancer (45.19%), ovarian cancer (26.23%), and endometrial cancer (28.57%). Nearly one-third of the patients (28.83%) received chemotherapy before surgery. Laparoscopic surgery constituted the primary surgical approach, representing 90.65% of all procedures. Further demographic and clinical characteristics are detailed in Table 4.

**Table 4:** Univariate analysis of factors associated with symptom subgroups.

Variables (*n*[%])	Total (*n* = 385)	C1 (*n* = 260)	C2 (*n* = 125)	Statistic	*P*
Age				*χ*^2^ = 10.83	.004
≥60	88 (22.86)	72 (27.69)	16 (12.80)		
40–59	241 (62.60)	154 (59.23)	87 (69.60)		
18–39	56 (14.55)	34 (13.08)	22 (17.60)		
Occupation				*χ*^2^ = 0.43	.512
Retired/unemployed	191 (49.61)	132 (50.77)	59 (47.20)		
Employed	194 (50.39)	128 (49.23)	66 (52.80)		
Education level				*χ*^2^ = 4.79	.188
Primary school or below	98 (25.45)	72 (27.69)	26 (20.80)		
Junior high school	106 (27.53)	72 (27.69)	34 (27.20)		
High school/technical secondary school	77 (20.00)	54 (20.77)	23 (18.40)		
College degree or above	104 (27.01)	62 (23.85)	42 (33.60)		
Marital status				–	.830
Married	355 (92.21)	238 (91.54)	117 (93.60)		
Unmarried	5 (1.30)	4 (1.54)	1 (0.80)		
Divorced/widowed	25 (6.49)	18 (6.92)	7 (5.60)		
Living arrangement				–	.836
Living with family	357 (92.73)	242 (93.08)	115 (92.00)		
Living alone	21 (5.45)	14 (5.38)	7 (5.60)		
Others	7 (1.82)	4 (1.54)	3 (2.40)		
Place of residence				*χ*^2^ = 1.04	.594
Urban	228 (59.22)	151 (58.08)	77 (61.60)		
Rural	55 (14.29)	36 (13.85)	19 (15.20)		
County/town	102 (26.49)	73 (28.08)	29 (23.20)		
Source of medical expenses				*χ*^2^ = 0.09	.764
Medical insurance	355 (92.21)	239 (91.92)	116 (92.80)		
Self-payment	30 (7.79)	21 (8.08)	9 (7.20)		
Do you have children				*χ*^2^ = 0.03	.864
No	29 (7.53)	20 (7.69)	9 (7.20)		
Yes	356 (92.47)	240 (92.31)	116 (92.80)		
Preoperative chemotherapy				*χ*^2^ = 87.64	<.001
No	274 (71.17)	224 (86.15)	50 (40.00)		
Yes	111 (28.83)	36 (13.85)	75 (60.00)		
Disease type				*χ*^2^ = 38.37	<.001
Cervical cancer	174 (45.19)	114 (43.85)	60 (48.00)		
Ovarian cancer	101 (26.23)	49 (18.85)	52 (41.60)		
Endometrial cancer	110 (28.57)	97 (37.31)	13 (10.40)		
Surgical approach				*χ*^2^ = 3.07	.080
Laparoscopy	349 (90.65)	231 (88.85)	118 (94.40)		
Open abdominal	36 (9.35)	29 (11.15)	7 (5.60)		

*χ*^2^: Chi-square test, -: Fisher exact (see variables: Marital status, Living arrangement).

### 3.2. Incidence and severity of symptoms

As presented in Table 1, sixteen symptoms had a prevalence rate of ≥20%, with the most common being pain (n = 373, 96.88%), fatigue (n = 372, 96.62%), and disturbed sleep (n = 351, 91.17%). In terms of symptom severity, fatigue (mean = 4.03, *SD* = 2.15), pain (mean = 4.01, *SD* = 2.21), and disturbed sleep (mean = 3.51, *SD* = 2.25) ranked among the most severe ones. QoL domain scores are presented in Table 2.

**Table 1:** Symptom prevalence and severity measured by the MDASI-PeriOp-GYN (*N* = 385).

Symptom	Prevalence *n* (%)	Severity score mean ± SD
Pain	373 (96.88)	4.01 ± 2.21
Fatigue	372 (96.62)	4.03 ± 2.15
Nausea	171 (44.42)	2.06 ± 2.62
Disturbed sleep	351 (91.17)	3.51 ± 2.25
Distress	302 (78.44)	3.03 ± 2.34
Shortness of breath	68 (17.66)	0.75 ± 1.76
Difficulty remembering	67 (17.40)	0.45 ± 1.42
Lack of appetite	313 (81.30)	2.64 ± 1.95
Drowsiness	249 (64.68)	2.14 ± 2.15
Dry mouth	157 (40.78)	1.55 ± 2.21
Sadness	255 (66.23)	2.76 ± 2.58
Vomiting	71 (18.44)	0.92 ± 2.16
Numbness/tingling	222 (57.66)	1.77 ± 2.02
Bloating	249 (64.68)	2.17 ± 2.29
Abdominal cramping	173 (44.94)	1.69 ± 2.22
Constipation	198 (51.43)	1.45 ± 1.85
Diarrhea	30 (7.79)	0.17 ± 0.78
Dizziness	245 (63.64)	2.10 ± 2.17
Confusion	230 (59.74)	1.99 ± 2.20
Hot flashes	249 (64.68)	2.04 ± 2.14
Urinary pain	51 (13.25)	0.57 ± 1.64
Difficulty urinating	39 (10.13)	0.43 ± 1.46

**Table 2:** Quality of life domain scores measured by the FACT-G (*N* = 385).

QoL domain	Score, mean ± SD
Physical well-being	14.56 ± 5.24
Social/family well-being	16.06 ± 7.19
Emotional well-being	14.25 ± 5.00
Functional well-being	13.11 ± 5.32
Total quality of life sco	57.99 ± 14.22

FACT-G = Functional Assessment of Cancer Therapy: General. Higher scores indicate better quality of life.

### 3.3. Latent profile analysis of symptoms and nomenclature of subgroups in patients after gynecologic malignancy surgery

A total of 5 latent profile models were fitted based on 16 symptoms, and the fit indices are presented in Table 3. The values of the information criteria decreased continuously with an increase in the number of classes, with the most pronounced reduction observed from 1-class to 2-class models. When 2 or 3 latent classes were retained, both the Lo-Mendell-Rubin test and the Bootstrapped likelihood ratio test yielded significant *P*-values, and entropy values were above 0.95, indicating that the 3-class model was statistically superior to the 2-class model. However, the selection of a latent class model should also consider the interpretability of the classes and the sample size requirements of subsequent analyses.^[24]^ In this study, symptom–QoL networks were planned to be constructed separately for each latent class, requiring adequate sample sizes to ensure stable and reliable estimation. In the 3-class model, the smallest class comprised only 48 patients (12.50%). Simulation evidence indicates that, with 20 nodes, this sample size is unlikely to meet the requirements for stable network edge weight and centrality estimation.^[25]^ In summary, the 2-class model was selected because, although its statistical fit was slightly lower than that of the 3-class model, it provided sufficient sample sizes in both classes to support reliable network estimation and clinically interpretable subgroup comparisons.

**Table 3:** Fit indices for latent profile analysis models of postoperative symptoms.

Model	AIC	BIC	aBIC	Entropy	LMR_*P*	BLRT_*P*	Category probability
1	27256.941	27383.445	27281.913	–	–	–	1.000
2	24879.629	25073.337	24917.867	0.952	.001	<.001	.675/.325
3	24085.167	24346.081	24136.672	0.953	.001	<.001	.551/.325/.125
4	23750.646	24078.766	23815.417	0.922	.596	<.001	.400/.262/.262/.075
5	23466.269	23861.594	23544.306	0.942	.177	<.001	.400/.273/.190/.073/.065

AIC = Akaike information criterion; BIC = Bayesian information criterion, aBIC = sample size-adjusted BIC, LMR_P = Lo-Mendell-Rubin adjusted LRT test *P*-value, BLRT_P = Bootstrap Likelihood Ratio Test *P*-value.

**Figure 1.**
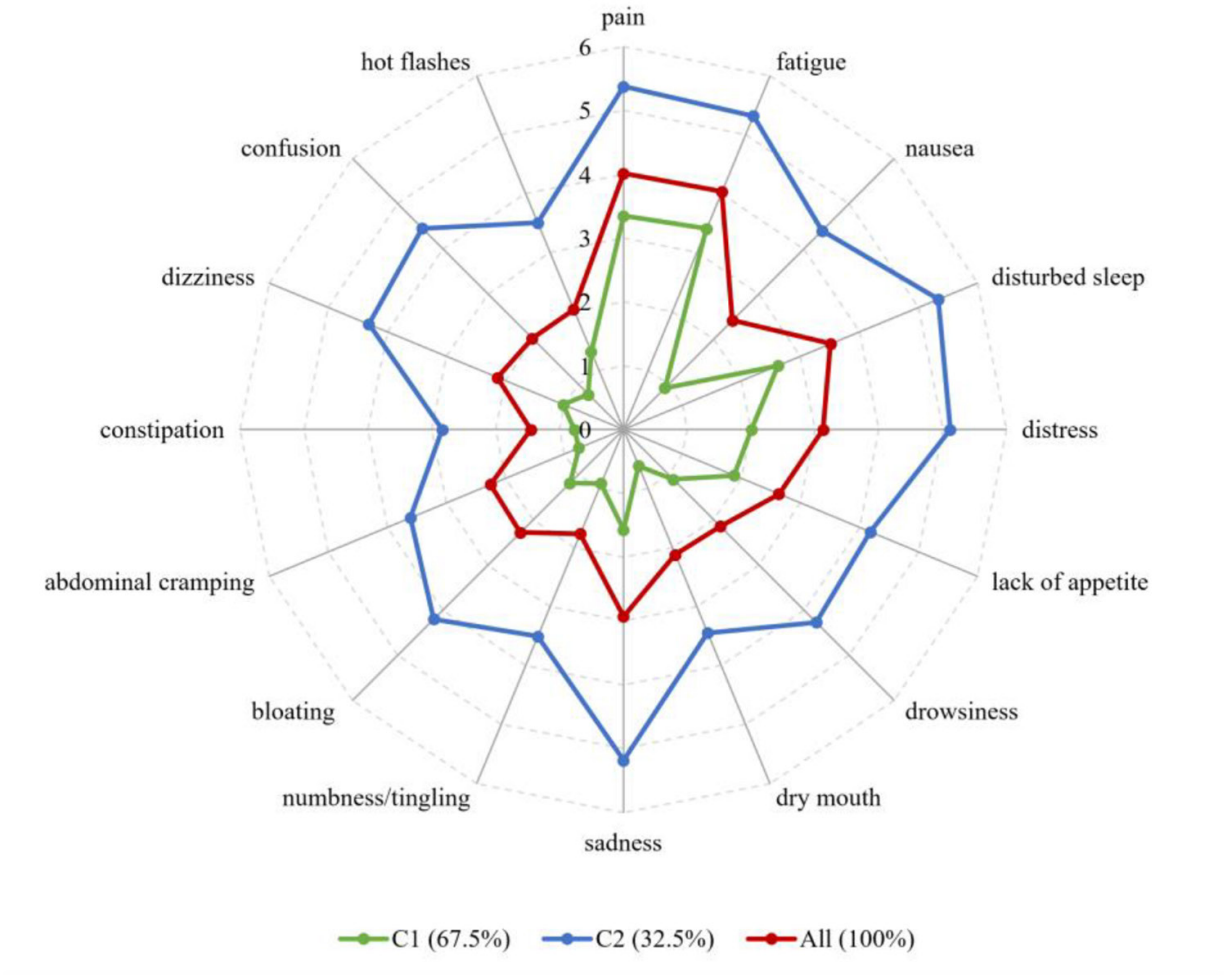
Distribution of postoperative symptom severity profiles.

The mean distribution of symptom severity in the 2 subgroups of patients with gynecologic malignancies is shown in Figure 1. Patients in Subgroup 1 (C1) exhibited lower severity across all symptoms than those in Subgroup 2 (C2), with a total severity score of 24.33 ± 13.09. Thus, C1 was named the low symptom burden group, comprising 260 patients, accounting for 67.53% of the total sample. Patients in Subgroup 2 demonstrated moderate-to-high symptom severity, with a total severity score of 69.31 ± 19.32. Accordingly, C2 was named the moderate-to-high symptom burden group, consisting of 125 patients, representing 32.47% of the total sample.

### 3.4. Univariate and multivariate analysis of potential postoperative symptom classes in patients with gynecological malignancies

Univariate analysis revealed statistically significant differences between the 2 groups in age, history of preoperative chemotherapy, and tumor type (*P* < .05), as shown in Table 4 and Supplementary Table S1, Supplemental Digital Content 2. These variables were included as independent variables in a multivariate logistic regression analysis, with age >60 years, receipt of preoperative chemotherapy, and cervical cancer set as reference categories. Multivariate analysis demonstrated that patients aged 40 to 59 years (odds ratio [OR] = 2.38, 95% CI: 1.19–4.74, *P* = .014), those who received preoperative chemotherapy (OR = 12.89, 95% CI: 6.54–25.41, *P* < .001), and patients with ovarian cancer (OR = 5.09, 95% CI: 2.55–10.13, *P* < .001) were more likely to be classified into group C2, as presented in Table 5. All GVIF^ (1/ (2 × *df*)) values were close to 1 (age: 1.008; preoperative chemotherapy: 1.116; disease type: 1.060), confirming no evidence of multicollinearity. The Hosmer–Lemeshow test was nonsignificant (*χ*^2^ = 57.05, *P* = .48), and the AUC was 0.80 (95% CI: 0.75–0.85), indicating adequate model fit. Firth penalized logistic regression yielded highly consistent results (Supplementary Table S2, Supplemental Digital Content 1), indicating that the observed associations were robust.

**Table 5:** Multivariate logistic regression analysis of factors associated with symptom subgroups.

Variables	*β*	*S.E*	*Z*	*P*	OR (95%CI)
Age (≥60 as reference)					
40–59	0.87	0.35	2.46	.014	2.38 (1.19–4.74)
18–39	0.87	0.45	1.95	.051	2.39 (1.00–5.72)
Preoperative chemotherapy (no as reference)					
Yes	2.56	0.35	7.38	<.001	12.89 (6.54–25.41)
Disease type (Cervical cancer as reference)					
Ovarian cancer	1.63	0.35	4.63	<.001	5.09 (2.55–10.13)
Endometrial cancer	0.18	0.43	0.42	.672	1.20 (0.52–2.77)

Categorical variables were coded as factors with specified reference categories (age: ≥60 years; preoperative chemotherapy: no; disease type: cervical cancer).

GVIF^(1/(2 × Df)) values: age, 1.008; preoperative chemotherapy, 1.116; disease type, 1.060.

Hosmer–Lemeshow test: *χ*^2^ = 57.05, *P* = .48. AUC = 0.80 (95% CI: 0.75–0.85).

Firth penalized logistic regression yielded consistent results (see Supplementary Table S2, Supplemental Digital Content 1).

### 3.5. Symptom–QoL network analysis in different subgroups of patients after surgery for gynecological malignancies

**Figure 2.**
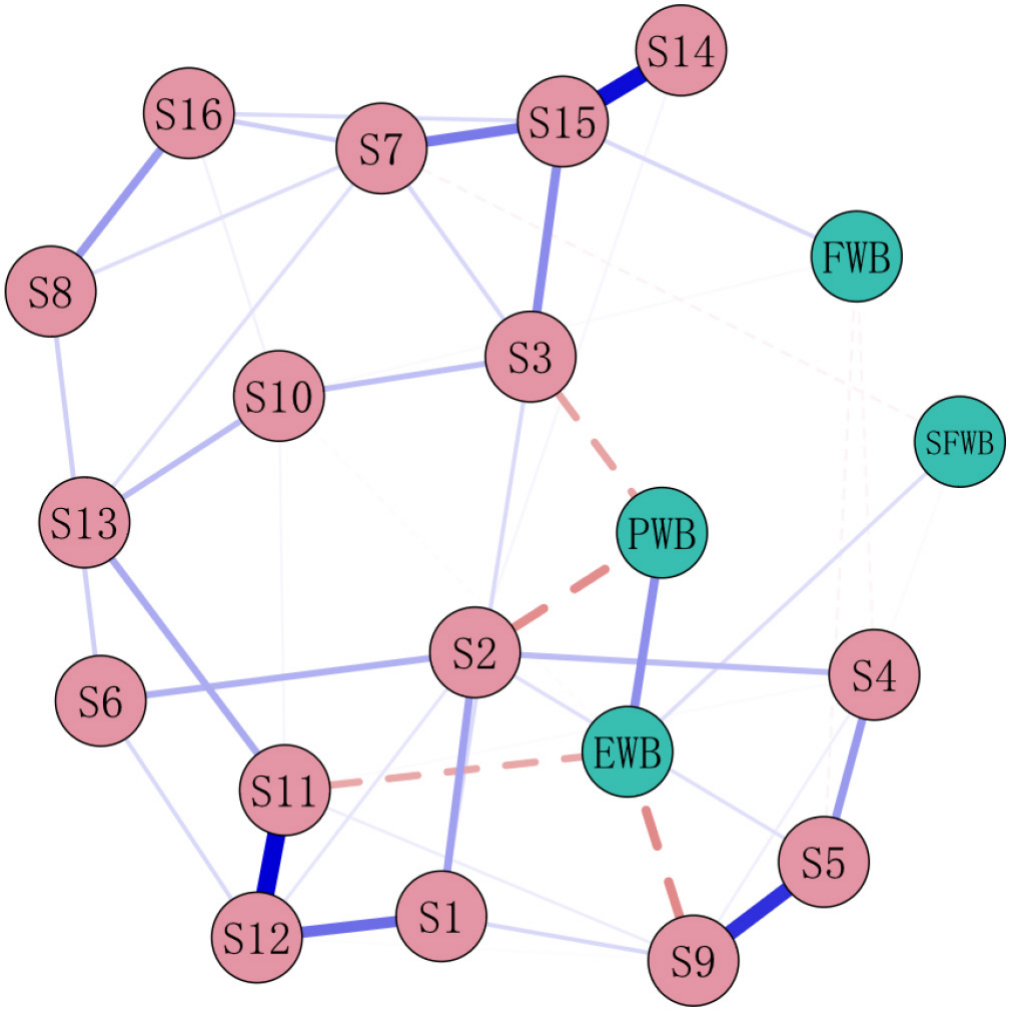
Symptom–QoL network for the low symptom burden group (C1). Red circles represent symptoms, and green circles represent quality of life domains. Solid blue lines indicate positive correlations, whereas dashed red lines indicate negative correlations. Edge width reflects the absolute magnitude of the edge weight. Networks were estimated using the EBICglasso algorithm (*γ* = 0.5). S1 = pain, S2 = fatigue, S3 = nausea, S4 = disturbed sleep, S5 = distress, S6 = lack of appetite, S7 = drowsiness, S8 = dry mouth, S9 = sadness, S10 = numbness/tingling, S11 = bloating, S12 = abdominal cramping, S13 = constipation, S14 = dizziness, S15 = confusion, = S16, hot flashes, EWB = emotional well-being, FWB = functional well-being, PWB = physical well-being, SFWB = social/family well-being.

**Figure 3.**
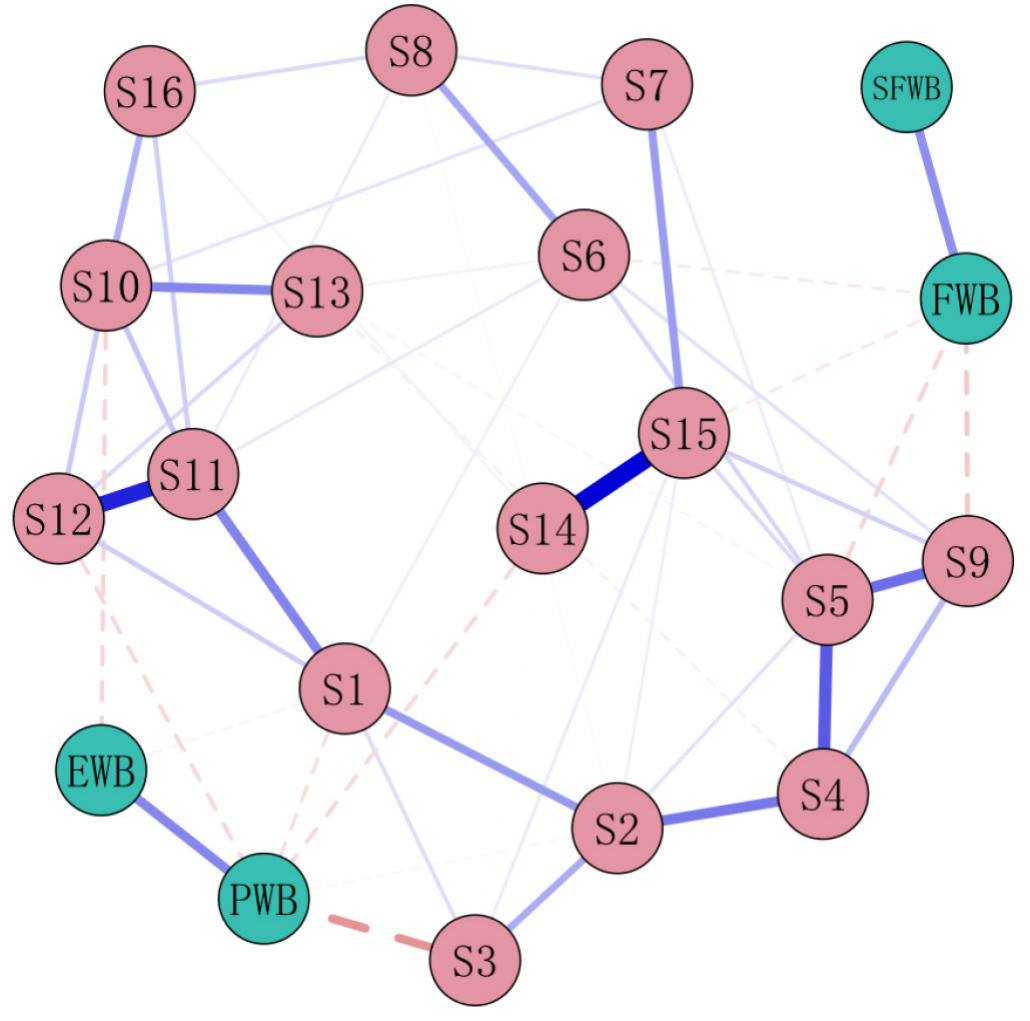
Symptom–QoL network for the moderate-to-high symptom burden group (C2). See Figure 2. for network visualization details. EWB = emotional well-being, FWB = functional well-being, PWB = physical well-being, SFWB = social/family well-being.

**Figure 4.**
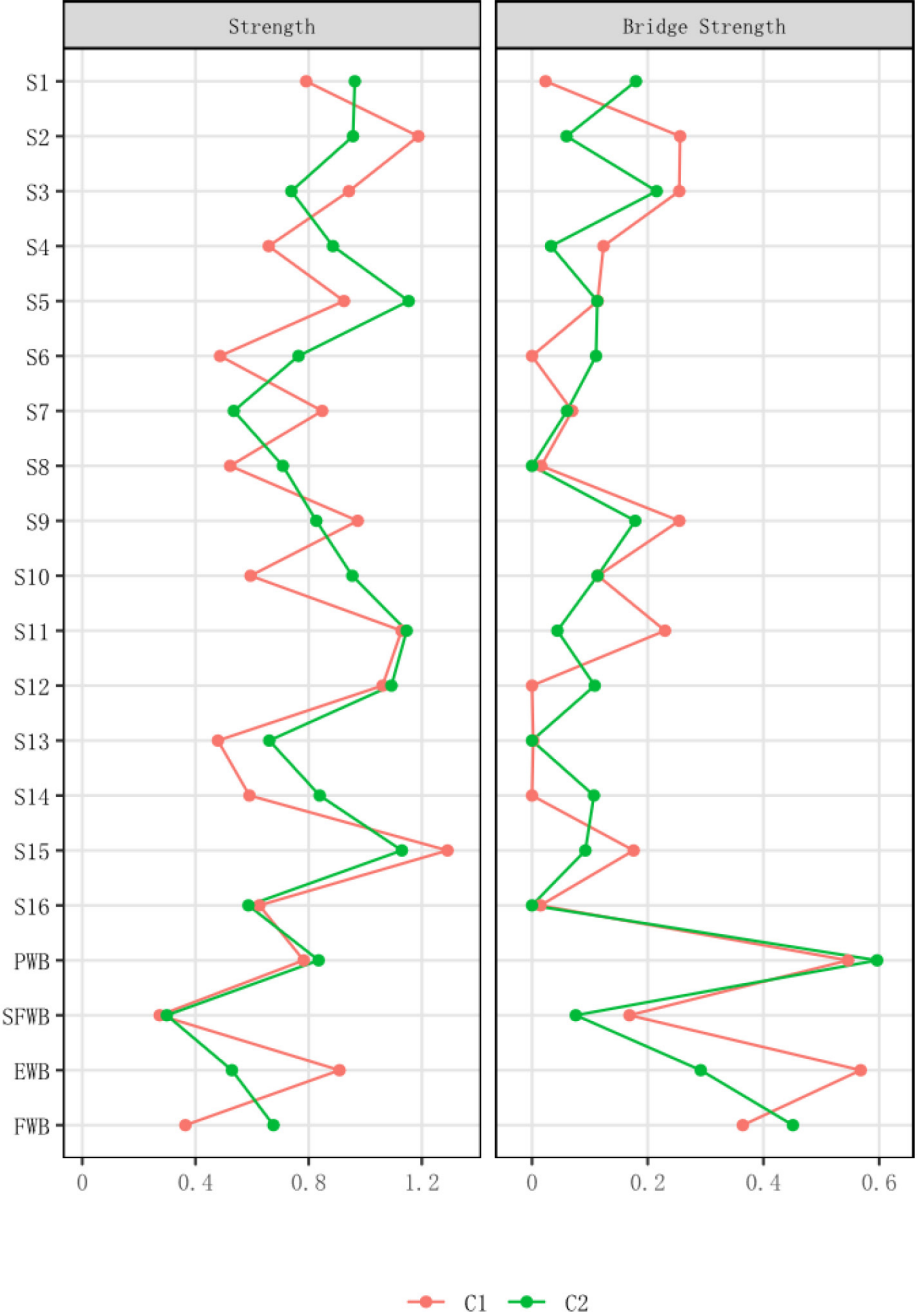
Strength and bridge strength centrality measures for C1 and C2.

**Figure 5.**
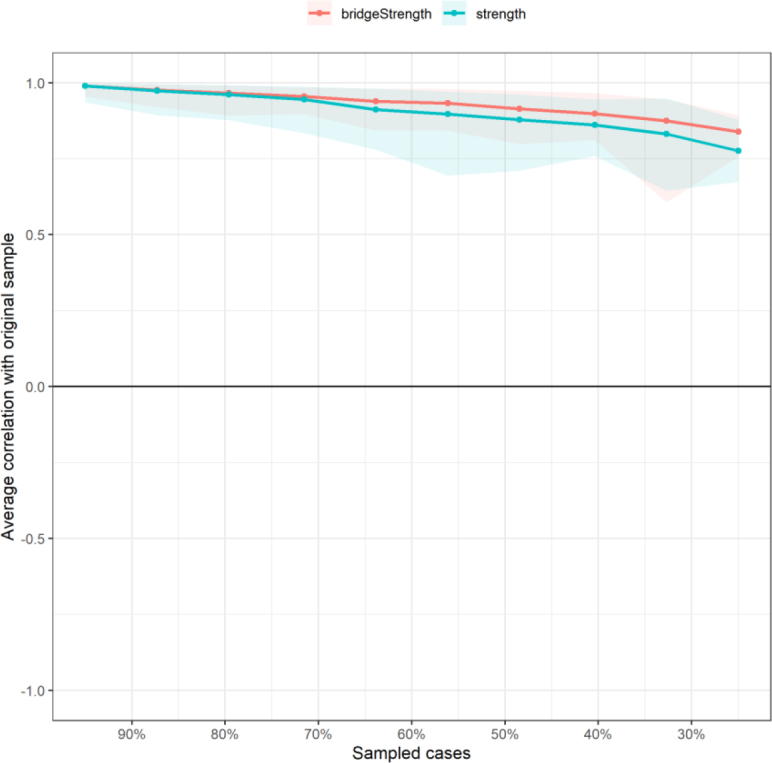
Correlation stability coefficient plots for the low symptom burden group (C1).

**Figure 6.**
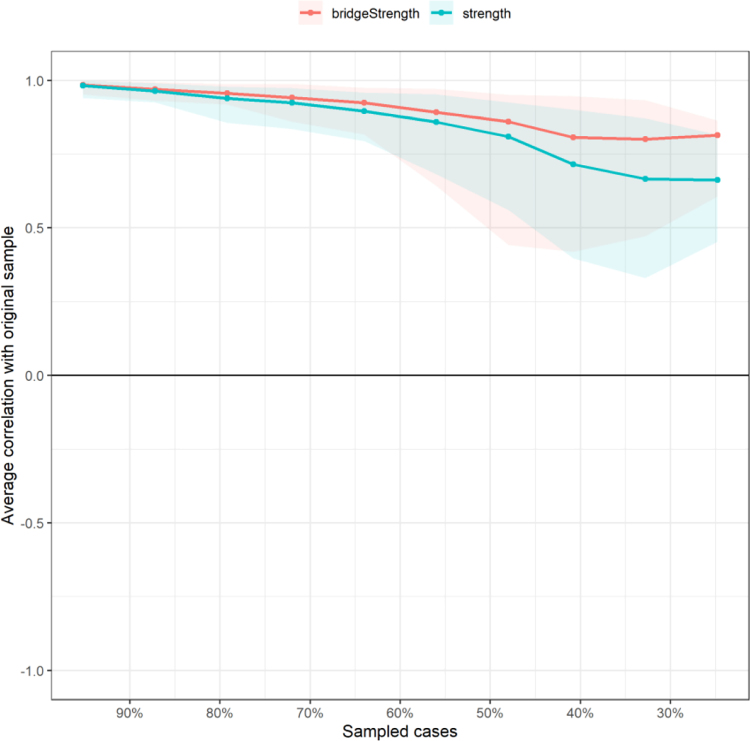
Correlation stability coefficient plots for the moderate-to-high symptom burden group (C2).

**Figure 7.**
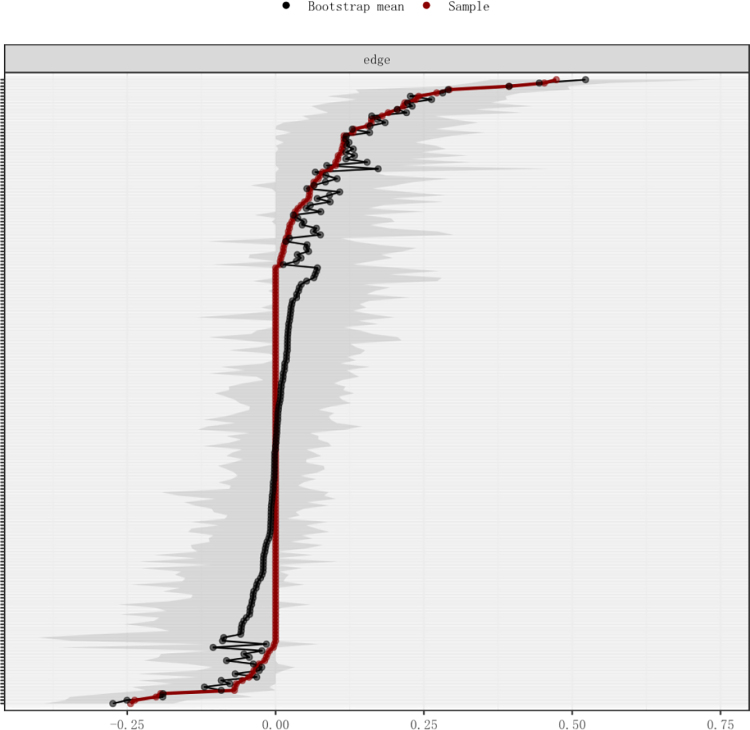
Accuracy of edge weights in the low symptom burden group (C1).

**Figure 8.**
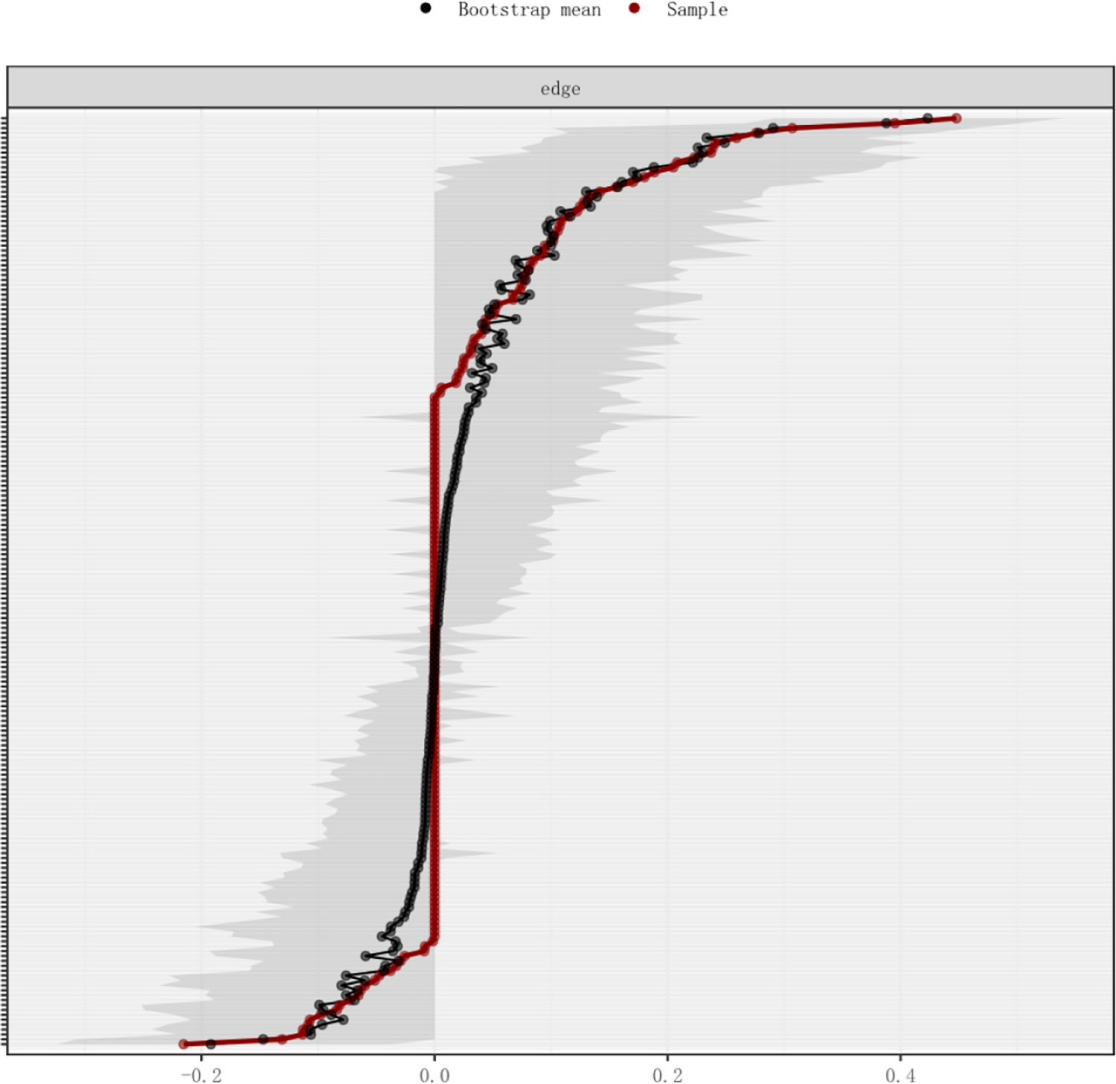
Accuracy of edge weights in the moderate-to-high symptom burden group (C2).

**Figure 9.**
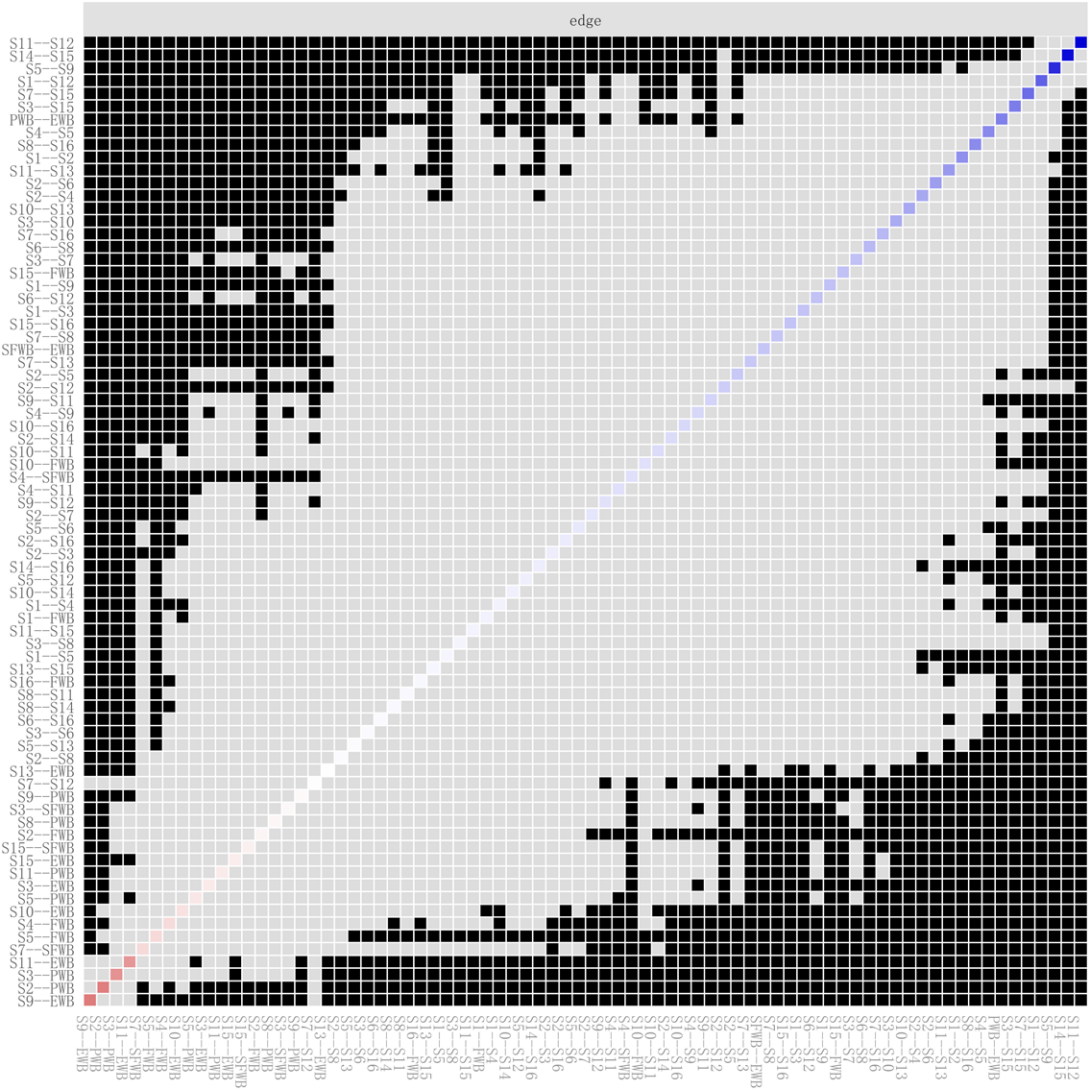
Bootstrapped difference test for edge weights in the low symptom burden group (C1). Black squares indicate significant differences between edge weights (*P* < .05, Bonferroni-corrected), whereas gray or white squares indicate nonsignificant differences. Edges are ordered according to absolute edge weight values.

**Figure 10.**
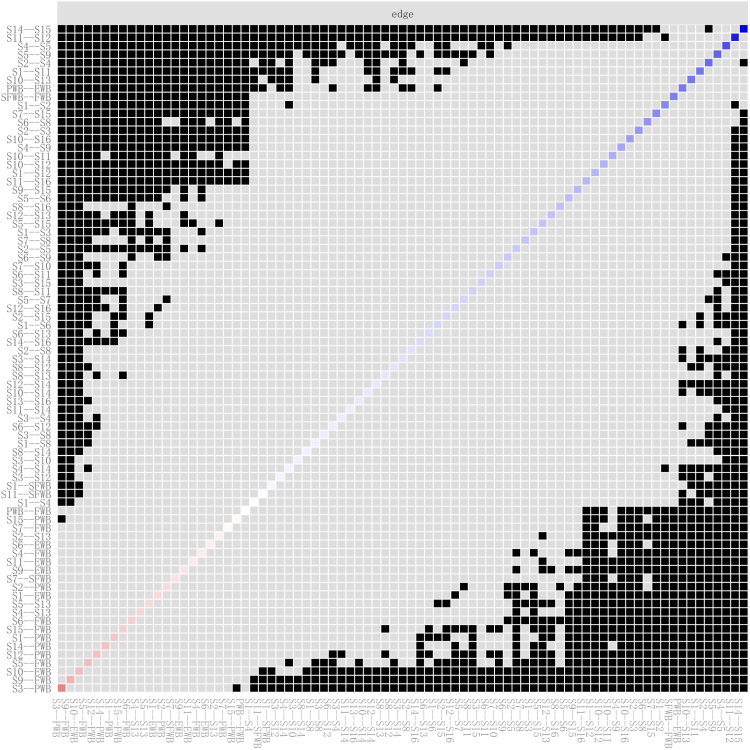
Bootstrapped difference test for edge weights in the moderate-to-high symptom burden group (C2). Black squares indicate significant differences between edge weights (*P* < .05, Bonferroni-corrected), whereas gray or white squares indicate nonsignificant differences. Edges are ordered according to absolute edge weight values.

The networks of potential subgroups in patients after surgery for gynecological malignancies are presented in Figures 2 and 3 (solid lines indicate positive correlations, and dashed lines indicate negative correlations). In the low symptom burden group (C1), the strongest positive correlation was observed between abdominal distension and abdominal cramping, while the strongest negative correlation was between sadness and emotional status. A relatively strong negative correlation was also found between fatigue and physical status. Compared with group C1, group C2 (characterized by moderate-to-high symptom burden) exhibited strengthened correlations among emotional symptoms, including sadness, distress, and disturbed sleep. The strongest negative correlation was observed between nausea and physical status. The node strength and bridge strength of the 2 subgroups are shown in Figure 4 (see also Supplementary Table S1, Supplemental Digital Content 2). In group C1, drowsiness (1.29), fatigue (1.19), and abdominal distension (1.13) exhibited the highest strength values and were identified as the core symptoms. In group C2, distress (1.15), abdominal distension (1.14), and drowsiness (1.13) represented the core symptoms. Bridge strength analysis revealed that emotional status (0.57) had the highest bridge strength in group C1, while physical status (0.60) had the highest bridge strength in group C2. Emotional status and physical status served as the key bridges linking symptoms and QoL in groups C1 and C2, respectively. The stability and edge accuracy of the symptom–QoL networks for the 2 subgroups are illustrated in Figures 5–8. The correlation stability coefficient (CS-coefficient) for strength centrality was 0.67 in the low symptom burden group (C1) and 0.44 in the moderate-to-high symptom burden group (C2). Both values exceeded the threshold of 0.25 for acceptable stability, and the C1 value exceeded 0.50, indicating good stability.^[20]^ Bootstrapped difference tests for edge weights indicated that the strongest edges in both networks were estimated with adequate precision and were significantly stronger than weaker connections (Figs. 9–10). Bootstrapped difference tests for node centrality indicated that the highest-ranking symptoms for strength (drowsiness and fatigue in C1; distress in C2) and bridge strength (emotional status in C1; physical status in C2) were statistically distinguishable from nodes with lower centrality (Supplementary Figures S1–S2, Supplemental Digital Content 3).

## 4. Discussion

### 4.1. Subgroup heterogeneity in postoperative symptoms

Using LPA, this study classified postoperative patients with gynecologic malignancies into 2 groups based on low and moderate-to-high symptom burden, revealing significant heterogeneity in their symptom experiences. Consistent with findings in gastrointestinal,^[26]^ breast,^[27]^ and head and neck cancer studies,^[28]^ our results confirm substantial symptom variability even among patients with the same disease and similar treatment stages, thereby supporting a paradigm shift from population-based to individualized symptom management. The low symptom burden group (C1) exhibited mild physical symptoms, whereas the moderate-to-high burden group (C2) presented with complex, severe symptoms, characterized by prominent emotional manifestations. Analogous to the “fatigue-dominant” symptom network reported by Röttgering et al in glioma patients, this suggests a synergistic co-occurrence of physical and emotional symptoms in individuals with a high burden. Stratified, subgroup-specific precision interventions may thus support more targeted resource allocation for this patient population.

The profiles identified in this study should be interpreted in the context of postoperative day 3, when most surgical symptoms reach their peak severity.^[13]^ Longitudinal evidence in gynecologic oncology indicates that symptom clusters are dynamic during the first postoperative week, with some clusters emerging or dissolving across time points.^[29]^ The relative centrality of individual symptoms may also shift as acute analgesic requirements decrease. Consequently, interventions derived from these network maps are most applicable to the first 72 hours post-surgery. Priorities for subacute and chronic recovery phases may differ and warrant investigation through longitudinal designs.

### 4.2. Predictors of the two categories

Results showed that age 40 to 59, preoperative chemotherapy, and ovarian cancer diagnosis were factors significantly associated with moderate-to-high symptom burden. The finding that middle-aged patients had higher symptom burden (OR = 2.38) may reflect greater sensitivity to symptoms, higher expectations for QoL, and more stress from family and social roles, all of which may increase psychological distress and exacerbate symptoms.^[30]^ Patients who received preoperative chemotherapy also had higher symptom burden (OR = 12.89), which may be related to the toxic effects of chemotherapy drugs, such as myelosuppression, gastrointestinal upset, fatigue, and nerve damage. These side effects can persist after surgery and may be exacerbated by surgical trauma.^[31]^ Ovarian cancer was associated with higher symptom burden (OR = 5.09), possibly reflecting late diagnosis, extensive surgeries (often involving multiple organ resections), frequent complications (such as ascites or bowel obstruction), and prolonged treatment courses involving multiple cycles of chemotherapy, all of which are associated with greater symptom burden.^[32]^ The type of surgery (laparoscopic or open) was not associated with symptom levels, possibly reflecting the high rate of laparoscopy and standardized pain management; this warrants further study with larger sample sizes. In summary, these findings suggest that clinical attention should be directed toward middle-aged patients, those who received preoperative chemotherapy, and those with ovarian cancer.

### 4.3. *Symptom–QoL network of subgroups*

#### 4.3.1. Low symptom burden group

Network characteristics of group C1 revealed that symptom experiences were dominated by physical symptoms, with abdominal distension, fatigue, and drowsiness as core manifestations. Postoperative abdominal distension is a specific clinical symptom in patients with gynecologic malignancies, associated with surgical scope, site, and use of analgesics: factors that often induce intestinal paralysis or even intestinal obstruction, severely impairing postoperative recovery.^[33]^ Fatigue and drowsiness may be related to anesthetic metabolism, postoperative pain management, and sleep rhythm disturbance; fatigue, a persistently prevalent symptom, occupies a central position in numerous studies on cancer-related symptoms.^[34]^ Sadness was negatively correlated with emotional status, suggesting that even mild physical symptoms may be associated with poorer QoL through emotional pathways.

Thus, interventions for the low symptom burden group should be based on physical symptom management supplemented by emotional support. Alleviating sadness and fatigue can improve patients’ emotional and physical conditions, thereby enhancing their QoL. Specific symptom management strategies are as follows: For abdominal distension, evidence-based dietary plans and enhanced recovery after surgery principles should be implemented,^[33]^ such as avoiding prolonged preoperative fasting and administering oral carbohydrate drinks 2 hours preoperatively; postoperatively, early bed exercises (e.g., hip lifting, turning over, ankle pump movements) are encouraged to promote flatus, while gas-producing foods are avoided; traditional Chinese medicine nursing techniques such as external application of Chinese herbs and acupoint sticking may be combined. For pain, drowsiness, and fatigue, patients should be guided to balance rest and activity, maintain adequate nutrition, and gradually perform rehabilitation exercises; meanwhile, multimodal analgesia strategies should be applied to reduce opioid dosage, which can alleviate drowsiness and fatigue. Emotional support should be integrated into routine care: distress thermometers can be used to screen for psychological and emotional issues,^[35]^ and disease-related health education or short-term psychological counseling should be provided to prevent the deterioration of mental health problems.

#### 4.3.2. Moderate-to-high symptom burden group

The symptom network of group C2 exhibited a more complex “psychosomatic comorbidity” pattern. Emotional symptoms, including sadness, distress, and disturbed sleep, demonstrated strong interconnections, forming a distinct “emotional distress subnetwork.” This subnetwork, anchored by the core node of distress, showed robust negative associations with physical symptoms (e.g., nausea, fatigue) and overall physical status, forming a pattern consistent with a “psychological-somatic-physical” feedback loop. Notably, although nausea was not among the symptoms with the highest strength centrality, it exhibited the strongest negative correlation with physical status: the bridge symptom connecting the symptom network to overall QoL in this subgroup. This suggests that nausea may play an important role in the co-occurrence of physical and psychological symptoms, and its management may contribute to preserving physical function and improving QoL. These findings corroborate previous evidence indicating a stronger link between psychological and physical symptoms in cancer patients experiencing severe symptom burden.^[36]^

The association between psychological distress and physical symptoms suggests that addressing emotional symptoms may be an important component of symptom management.^[37]^ The symptom experience of the moderate-to-high burden group is characterized by co-occurring psychological and physical symptoms, and these patterns are associated with elevated psychological distress. Distress emerged as the strongest core symptom in group C2, suggesting that psychological interventions may warrant consideration as a central component of symptom management. Concurrently, the centrality of nausea in the network indicates that its management may help reduce the combined burden of physical and psychological symptoms.

For emotional management, strategies such as cognitive behavioral therapy and Mindfulness-Based Stress Reduction should be considered; these approaches may alleviate emotional suffering and improve sleep quality, reduce fatigue, and enhance physical function. Furthermore, developing a culturally adapted, trauma-informed nursing program for gynecologic oncology patients may assist healthcare providers in identifying psychological issues arising during cancer diagnosis, treatment, and care, thereby facilitating the formulation of targeted interventions.^[38]^ Regarding nausea management, in addition to conventional pharmacotherapy, non-pharmacological approaches (e.g., acupoint stimulation, aromatherapy, and ginger consumption) should be integrated to implement comprehensive interventions.

## 5. Conclusion

Using LPA, this study categorized postoperative gynecological malignancy patients into low and moderate-to-high symptom burden groups during the acute postoperative phase (day 3). Symptom network analysis revealed differences in core and bridge symptoms between groups. The low burden group featured somatic core symptoms and emotional status as the bridge. The moderate-to-high burden group exhibited a “psychological-somatic” pattern with physical status as the central bridge. These findings provide a reference for symptom management strategies tailored to patient subgroups in the early postoperative period. Future longitudinal studies are needed to determine whether these subgroup and network characteristics persist at later recovery stages.

## 6. Limitations

First, symptom assessment was conducted on postoperative day 3, within the acute recovery phase. Several time-specific factors may have shaped the observed symptom profiles. Most patients received opioid-based analgesia, standard during the first 48 to 72 hours postoperatively; opioid use is known to be associated with nausea, vomiting, and drowsiness.^[39]^ The prominence of drowsiness and nausea as core symptoms in both subgroups may therefore partially reflect pharmacological side effects rather than disease- or surgery-related burden alone. Concurrently, the systemic inflammatory response to surgical trauma is associated with fatigue, pain, and sleep disturbance: symptoms also among the most severe in our sample and known to often peak around postoperative day 3. Longitudinal studies in gynecologic oncology patients further demonstrate that symptom clusters undergo reconfiguration during the first postoperative week,^[29]^ indicating that the network structures reported here are specific to this early time point. Our findings therefore characterize the acute postoperative phase and may not directly generalize to later stages of recovery.

Second, several design-related factors should be considered when interpreting these results. The use of a >20% incidence threshold to select symptoms for the LPA, while methodologically grounded, is inherently a pragmatic choice and may have excluded less prevalent but clinically relevant symptoms. The cross-sectional design precludes causal inference among symptoms, and the absence of biological indicators limits mechanistic exploration. In addition, convenience sampling from a single tertiary hospital may limit generalizability. Future studies with larger, multicenter samples and longitudinal designs are needed to validate these findings and evaluate the impact of different methodological choices on symptom network characteristics.

## Author contributions

**Data curation:** Wenting Liu.

**Writing – original draft:** Wenting Liu, Juan He, Han Chen, Haiyan An, Lu Jiang.

**Investigation:** Juan He.

**Validation:** Han Chen.

**Supervision:** Haiyan An.

**Funding acquisition:** Lu Jiang.

**Methodology:** Lu Jiang.








