# Supplementary material for: Symptoms and quality of life in gynecological cancer patients after surgery: Application of latent profile and network analysis
Source: Medicine (Baltimore). 2026 Jun 26;105(26):e49482. doi: 10.1097/MD.0000000000049482 (PMC13313711; doi:10.1097/MD.0000000000049482)
Supplement: Supplementary file 1 [file medi-105-e49482-s001.docx]

**Supplementary Table S2. Parameter estimates from standard and Firth penalized logistic regression models.**

| Variables | Standard logistic regression | | Firth Logistic Regression | |
| --- | --- | --- | --- | --- |
|  | OR (95%CI) | *P* | OR (95%CI) | *P* |
| Age (≥60 as reference) |  |  |  |  |
| 40-59 | 2.38 (1.19–4.74) | 0.014 | 2.31 (1.19–4.68) | 0.012 |
| 18-39 | 2.39 (1.00–5.72) | 0.051 | 2.34 (0.99–5.60) | 0.052 |
| Preoperative Chemotherapy (no as reference) |  |  |  |  |
| Yes | 12.89 (6.54–25.41) | <0.001 | 12.13 (6.41–24.50) | <0.001 |
| Disease type (Cervical cancer as reference) |  |  |  |  |
| Ovarian cancer | 5.09 (2.55–10.13) | <0.001 | 4.88 (2.54–9.94) | <0.001 |
| Endometrial cancer | 1.20 (0.52–2.77) | 0.672 | 1.19 (0.52–2.72) | 0.679 |

Note. No separation was detected. Firth regression was performed as a sensitivity analysis and yielded results consistent with the standard logistic regression model.
