# Supplementary material for: Symptoms and quality of life in gynecological cancer patients after surgery: Application of latent profile and network analysis
Source: Medicine (Baltimore). 2026 Jun 26;105(26):e49482. doi: 10.1097/MD.0000000000049482 (PMC13313711; doi:10.1097/MD.0000000000049482)
Supplement: Supplementary file 2 [file medi-105-e49482-s002.docx]

**Supplementary Table S1. Generalized variance inflation factor (GVIF) diagnostics for multicollinearity assessment.**

| variables | GVIF | Df | GVIF^(1/(2*Df)) |
| --- | --- | --- | --- |
|  |  |  |  |
| Age | 1.033 | 2 | 1.008 |
| Preoperative Chemotherapy | 1.245 | 1 | 1.116 |
| Disease type | 1.265 | 2 | 1.060 |
