# Supplementary figures and images for: Symptoms and quality of life in gynecological cancer patients after surgery: Application of latent profile and network analysis
Source: Medicine (Baltimore). 2026 Jun 26;105(26):e49482. doi: 10.1097/MD.0000000000049482 (PMC13313711; doi:10.1097/MD.0000000000049482)

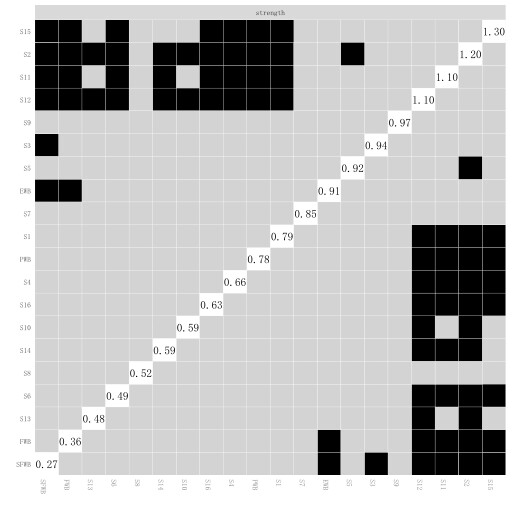

Supplement: Supplementary file 3 [file medi-105-e49482-s003.jpg]

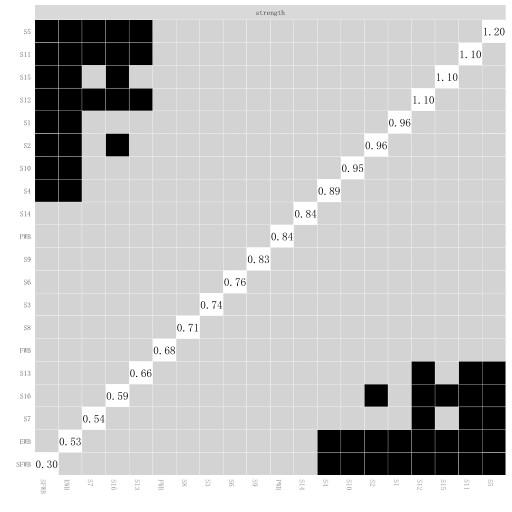

Supplement: Supplementary file 4 [file medi-105-e49482-s004.jpg]
